# Prevalence of high-riding vertebral arteries and narrow C2 pedicles among Central-European population: a computed tomography-based study

**DOI:** 10.1007/s10143-021-01493-6

**Published:** 2021-02-09

**Authors:** Tomasz Klepinowski, Natalia Żyłka, Bartłomiej Pala, Wojciech Poncyljusz, Leszek Sagan

**Affiliations:** 1 Department of Neurosurgery, Pomeranian Medical University Hospital No. 1, Szczecin, Poland; 2 Department of Diagnostic Imaging and Interventional Radiology, Pomeranian Medical University Hospital No. 1, Szczecin, Poland

**Keywords:** High-riding vertebral artery, Narrow pedicles, C2 isthmus, Craniocervical fusion, Prevalence

## Abstract

High-riding vertebral artery (HRVA) and narrow C2 pedicles (C2P) pose a great risk of injuring the vessel during C2 pedicle or transarticular screw placement. Recent meta-analysis revealed a paucity of European studies regarding measurements and prevalence of these anatomical variants. Three hundred eighty-three consecutive cervical spine CT scans with 766 potential screw insertion sites were analyzed independently by two trained observers. C2 internal height (C2InH), C2 isthmus height (C2IsH), and C2P width were measured. Kappa statistics for inter- and intraobserver reliability as well as for inter-software agreement were calculated. HRVA was defined as C2IsH of ≤ 5 mm and/or C2InH of ≤ 2 mm. Narrow C2P was defined as C2P width ≤ 4 mm. STROBE checklist was followed. At least 1 HRVA was found in 25,3% (95% CI 21,1–29,8) of patients (16,7% of potential sites). At least 1 narrow C2P was seen in 36,8% (95% CI 32,1–41,7) of patients (23,8% of potential sites). Among those with HRVA, unilateral HRVA was present in 68,0% (95% CI 58,4–77,0), whereas bilateral HRVA in 32,0% (95% CI 23,0–41,6). No difference in terms of laterality (right or left) was seen neither for HRVA nor narrow C2P. Significant differences were found between females and males for all measurements. Each parameter showed either good or excellent inter- or intraobserver, and inter-software agreement coefficients. HRVA and narrow C2P are common findings in Central-European population and should be appreciated at the planning stage before craniocervical instrumentation. Measurements can be consistently reproduced by various observers at varying intervals using different software.

## Introduction

Craniocervical junction (CCJ) is considered a tiger land in neurosurgery. Proximity of vitally important structures makes it difficult to approach. If instrumentation is planned, meticulous attention is paid to the course of vertebral arteries (VAs) and to the width of C2 pedicles. VA course presents many anatomical variations, some of them particularly affecting choice and outcome of craniocervical fusion [8]. A high-riding vertebral artery (HRVA) has been defined as a C2 isthmus height (C2IsH) of ≤ 5 mm and/or C2 internal height (C2InH) of ≤ 2 mm at the level 3 mm lateral to the border of the spinal canal [10, 11]. The presence of this anomaly increases the risk of vascular injury during CCJ instrumentation, far lateral, or extreme lateral approaches to the CCJ [2, 3]. Recent meta-analysis of global HRVA prevalence highlighted gaps in the literature as 16 of 20 analyzed studies came from Asia and only two were of European origin [8]. Given the fact that body height and genetics differ significantly between Asian and European subjects, it is rationalized to hypothesize that craniocervical and cervical measurements might be distinct in these populations [4, 13]. Prevalence of ossification of posterior longitudinal ligament is a good example of such discrepancy in cervical spine between the aforementioned regions [5]. Hence, HRVA and narrow C2 pedicle incidence should be specified in various human races. Therefore, this study aims to provide a response to that relevant remark, aiding the body of neurosurgical literature.

## Methodology

### Study design

A retrospective observational study of 383 consecutive anonymized computed tomography (CT) scans meeting inclusion criteria was analyzed by two independent observers. All the scans were made due to medical indications, none solely for the sake of this research. STROBE (Strengthening the Reporting of Observational Studies in Epidemiology) checklist was followed to ensure the correct structure. Each item of the STROBE was addressed.

### Sample size estimation

Assuming probability of type I error at 5% (α = 0,05) and the desired type II error at 20% (β = 0,2) with statistical power of the study being arbitrarily set at 80%, the minimum sample size has been estimated at 383 subjects (766 potential screw insertion sites) in order to be representative of the general thirty-eight million people Polish population in the region of Central Europe.

### Patient selection

Inclusion criteria were as follows: (1) adults, (2) CT scan of the cervical spine with adequate visualization of the atlas and axis, and (3) correct reformatting of the scans into the sagittal section. On the other hand, patients were excluded from the study in case of (1) instrumentation of the upper cervical spine, (2) any fracture of the C2 vertebra, (3) age less than 18 years, (4) inadequate quality of the study due to excessive motion or other technical issues, and (5) rheumatoid arthritis.

### Software and measurements

**Fig. 1:**
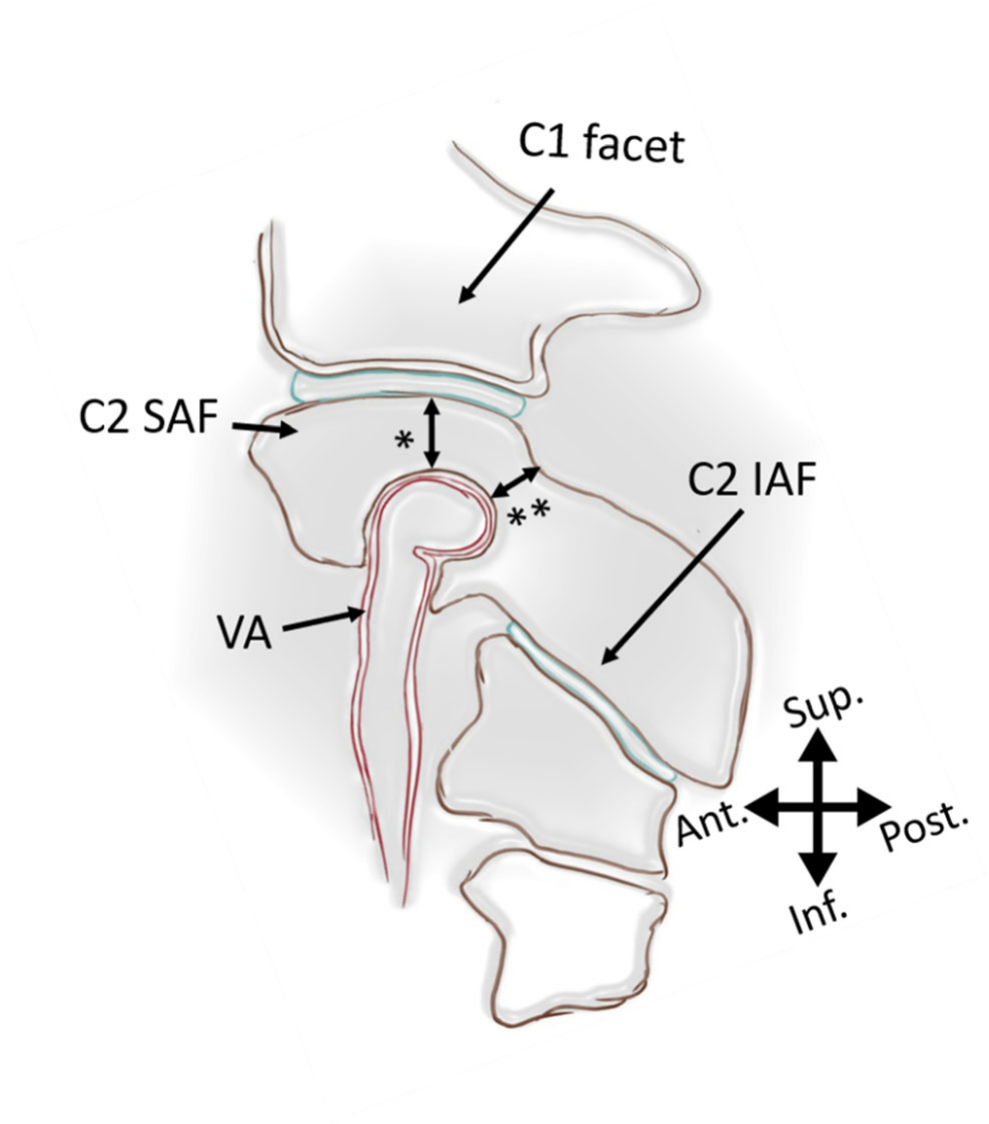
Rendition of the sagittal scan through atlantoaxial facetal joint showing measurements of the C2 internal height (*) and C2 isthmus height (**) that are used in the definition of the high-riding vertebral artery. Open access figure from Tomasz Klepinowski et al 2020 [1]. Original Publisher: Springer Nature (Creative Commons Attribution 4.0 International License http://creativecommons.org/licenses/by/4.0/)

**Fig. 2:**
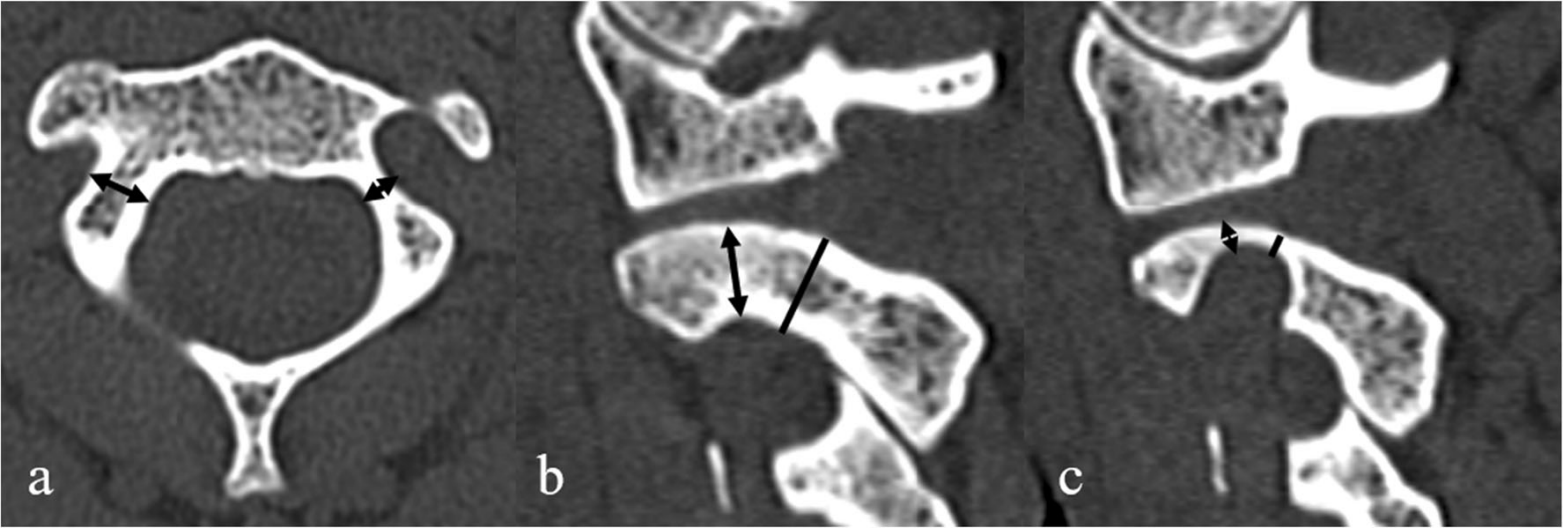
**a** Exemplary measurements of C2 pedicle width. Normal width on the right side. Narrow pedicle on the left side. **b** Normal C2 isthmus height (solid line) and C2 internal height (arrow line). **c** A side with a high-riding vertebral artery. Short C2 isthmus height (solid line). Short C2 internal height (arrow line)

All CT scans were obtained using Somatom Sensation 64 (Siemens Healthineers, Erlangen, Germany). Syn.govia (Siemens Healthineers, Erlangen, Germany) was employed for the primary measurements. Sagittal imaging was utilized for HRVA identification, whereas axial scans were used for pedicle appreciation. HRVA is declared present if C2IsH was ≤ 5 mm and/or C2InH was ≤ 2 mm measured 3 mm lateral to the border of the spinal canal (Fig. 1 and Fig. 2). Pedicles are deemed narrow if their width was ≤ 4 mm (Fig. 2). OsiriX MD 11.0 (Pixmeo SARL, Bernex, Switzerland) was used for secondary measurements in order to establish inter-software agreement. In order to determine whether rescue laminar screws are an option for subjects with bilateral HRVA or/and bilateral NP, laminar thickness was measured only in those individuals. Thin C2 lamina was defined as laminar thickness < 4 mm measured on axial CT scans.

### Interobserver, intraobserver, and inter-software variabilities

In order to determine interobserver variability, investigator triangulation was employed: two trained observers (neurosurgical resident TK and radiologic resident NŻ) were presented with a uniform PowerPoint slideshow (Microsoft, Redmond, USA) illustrating measurement methodology as well as caveats of possibly difficult cases to ensure harmonious technique. The slideshow was ended with a preliminary test of ten exemplary cases, followed by debriefing and a question-and-answer session afterward. For quantitative assessment of interobserver reproducibility, kappa statistic (κ_1_) evaluating agreement between TK and NŻ was calculated. On the other hand, intraobserver variability was established by re-measuring cases after a period of two to three months. For this, another kappa statistic (κ_2_) was calculated. For estimation of inter-software agreement coefficient, the same cases were re-measured using another software—OsiriX MD 11.0 (Pixmeo SARL, Bernex, Switzerland).

### Statistical analysis

Statistical analysis was performed by means of Statistica 13.3.0, TIBCO Software Inc. (Palo Alto, California, USA) and MetaXL 5.3 (EpiGear International Pty., Ltd., Brisbane, Australia). The level of significance for the *P* value of the comparative tests was arbitrarily set to < 5% (*P* < 0.05). To check for normality, a Shapiro-Wilk test was used. Welch’s *t* test was employed to determine variances. Pearson’s chi-square test was applied for categorical variables, whereas for continuous variables Student *t* for independent samples (in normal distribution) and Wilcoxon-Mann-Whitney (in non-normal distributions) tests were used. Possible values for kappa are within a range from – 1 to 1, with 0 tantamount to randomness, and 1 suggesting perfect reproducibility. As generally accepted, interpretation of the kappa values was as follows: < 0.20 poor agreement, 0.21–0.40 fair, 0.41–0.60 moderate, 0.61–0.80 good, and > 0.80 excellent agreement.

## Results

### Subject characteristics

A total of 383 cervical spine CT scans were analyzed comprising 766 potential screw insertion sites. There were 237 females (61,9%) and 146 males (38,1%). Mean age of the cohort was 43,2 years (range 22–86 years). As determined by eligibility criteria, none of the subjects had neither fractures within craniocervical junction, past procedure with cervical instrumentation, nor rheumatoid arthritis.

### Interobserver, intraobserver, and inter-software reliability coefficients

Assessment revealed that interobserver reliability of the left HRVA, right HRVA, left pedicle width, and right pedicle width was excellent (κ = 0,848), excellent (κ = 0,873), good (κ = 0,784), and good (κ = 0,784), respectively. Intraobserver reliability was found excellent for all four measurements with the following kappa statistics: κ = 0,852, κ = 0,834, κ = 0,864, and κ = 0,912 for the left HRVA, right HRVA, left pedicle width, and right pedicle width, respectively. Inter-software agreement between Syn.govia and OsiriX was excellent for the left HRVA (κ = 0,940), good for the right HRVA (κ = 0,703), excellent for the left pedicle width (κ = 0,896), and good for the right pedicle width (κ = 0,774). Summary of the agreement evaluation is presented in Table 1.

**Table 1:** Interobserver, intraobserver, and inter-software agreement coefficients

Measurement	Cohen’s kappa statistic (κ)	Reliability category*
Interobserver		
Left HRVA	0.848	Excellent
Right HRVA	0.873	Excellent
Left narrow C2P	0.784	Good
Right narrow C2P	0.795	Good
Intraobserver		
Left HRVA	0.852	Excellent
Right HRVA	0.834	Excellent
Left narrow C2P	0.864	Excellent
Right narrow C2P	0.912	Excellent
Inter-software		
Left HRVA	0.940	Excellent
Right HRVA	0.703	Good
Left narrow C2P	0.896	Excellent
Right narrow C2P	0.774	Good

*reliability categories were defined as follows: < 0.20 poor agreement, 0.21–0.40 fair, 0.41–0.60 moderate, 0.61–0.80 good, and > 0.80 excellent agreement. *HRVA*, high-riding vertebral artery. *C2P*, C2 pedicle

### Measurements and prevalence of high-riding vertebral arteries and narrow pedicles

Mean left C2IsH was 7,4 mm (SD ± 2,4 mm). Mean right C2IsH was 7,9 mm (SD ± 2,5 mm). Mean left C2InH was 6,7 mm (SD ± 2,6 mm). Mean right C2InH was 7,4 mm (SD ± 2,7). Mean left C2P width was 5 mm (SD ± 1,5 mm). Mean right C2P width was 5,4 mm (SD ± 1,6 mm). Prevalence of at least one HRVA was calculated to be 25,3% (95% CI 21,1–29,8) of subjects and 16,7% (95% CI 14,2–19,4) of potential screw insertion sites. Among individuals with HRVA, unilateral HRVA was present in 68,0% (95% CI 58,4–77,0), whereas bilateral HRVA was seen in 32,0% (95% CI 23,0–41,6). There was no statistically significant difference in terms of laterality: the left side showed prevalence of 56,6% (95% CI 47,9–65,1), and the right was found in 43,4% (95% CI 34,9–52,1). In male, at least 1 HRVA was seen in 16,4% (95% CI 10,8–22,9), whereas in female in 30,8% (95% CI 25,1–36,8). Prevalence of at least one C2 narrow pedicle was 36,8% (95% CI 32,1–41,7) of patients and 23,8% (95% CI 20,8–26,8) of potential screw insertion sites. Unilateral NP was present in 70,9% (95% CI 63,1–78,2), whereas bilateral NP was found in 29,1% (95% CI 21,8–36,9). Among those with NP, there was no significant side predilection: left 55,4% (95% CI 48,2–62,6) vs right 44,6% (37,4–51,8). As for gender predisposition, 30,8% (95% CI 23,6–38,6) of men and 40,5% (95% CI 34,3–46,8) presented NP. Relevant mean values of measurements in men and women differ significantly (Table 2).

**Table 2:** Comparison of mean measurements between males and females

Measurement	Male [mm]	Female [mm]	*P*
Left C2IsH	8,3	6,2	< 0,001
Left C2InH	7,5	6,9	< 0,001
Right C2IsH	8,8	7,4	0,02
Right C2InH	8,2	6,9	< 0,001
Left C2 pedicle width	5,4	4,8	< 0,001
Right C2 pedicle width	5,7	5,2	< 0,001

*C2IsH* C2 isthmus height. *C2InH* C2 internal height

#### Laminar thickness measurements for rescue screws

Among the subjects with bilateral HRVA, mean thickness of the left lamina was 5,48 mm (SD ± 1,21) on the left and 5,33 mm (SD ± 0,97) on the right with only four (13,3%) and three (10%) being thin (< 4 mm), respectively. In subjects with bilateral NP, mean left C2 laminar thickness was 5,49 mm (SD ± 1,06), whereas for the right, it was 5,45 mm (SD ± 0,83) with only 3 (7,3%) and 1 (2,4%) being thin, respectively.

## Discussion

The results of this CT-based study confirm that high-riding vertebral arteries and narrow C2 pedicles are relatively common. As global prevalence of HRVA has been estimated at 20,9% (95% CI 16,5–25,8%), the findings of this paper fit into the given confidence interval. Global prevalence of narrow C2P has not been studied, yet. However, its prevalence that was delineated here appears to be higher than showed by Asian researchers (Yeom et al in 2013) among Korean population (23,8% vs 9,5% of potential screw insertion sites). Possible explanation might be regional ethnical differences in the anatomy of high cervical spine [4].

### Updated European prevalence of HRVA

**Fig. 3:**
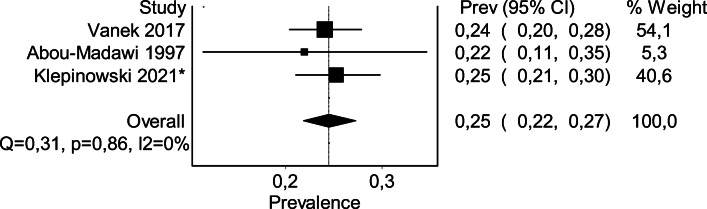
A forest plot of the updated European prevalence of the high-riding vertebral artery. *this study

Klepinowski et al. in 2020 conducted a comprehensive meta-analysis which presented global prevalence of HRVA. However, the regional prevalence analysis was of a limited value because of the small number of European studies. In fact, only two European papers were found eligible at that time, with one greatly outweighing the other. Therefore, the authors decided to aid the body of neurosurgical literature and proceed with the high-quality study. Including the present paper (Klepinowski 2021*), the updated European prevalence of at least one HRVA among a total of 944 subjects [1, 12] is now estimated at 24,52% (95% CI 21,83–27,32, *I*^2^ = 0,0 [0–32,7], Cochran Q = 0,31; random-effects model) (see Fig. 3). Comparing to the previously estimated prevalence, the updated one has narrower confidence interval, and the weight is more evenly distributed across the studies.

### Clinical implications

As it was recently noted, the most commonly employed method of managing both post-traumatic and rheumatic craniovertebral dislocation is Goel-Harms C1 lateral mass-C2 pedicle screw placement [6, 7]. This procedure has become a gold standard thanks to polyaxial screws that provide a solid bicortical purchase. Yet it is imperative to pay close attention preoperatively to two critical aspects: course of vertebral arteries and width of C2 pedicles. Practical guidelines for choice of craniocervical fusion method in case of HRVA have been published by the authors elsewhere [8]. The same pertains to the presence of C2 narrow pedicles, which may preclude safe screw placement. In those instances, rescue translaminar screws, laminar hooks, or spinal navigation might come in handy. As shown, most of the subjects even with bilateral HRVA or NP have normal C2 laminar thickness (≥ 4 mm), indicating that there usually exists a viable rescue option. Moreover, if HRVA or NP is identified early on the preoperative planning stage, additional steps might be taken to reduce a risk of injuring the VA. For instance, one may utilize intraoperative Doppler ultrasound as was elegantly demonstrated by Lofrese et al [9]. This can smoothly guide a spine surgeon through the procedure by delineating safe and danger zones where the VA could be inadvertently damaged. Therefore, combining the new information from the present study with already existing literature may further increase safety of the C1-C2 fusion and improve the overall outcome.

### Limitations

Although executed with attention to details and focused on quality, this study is not free from limitations. In spite of the intraobserver reliability being excellent across all the measurements, the interobserver and inter-software agreement coefficients were only good for both pedicle sides (interobserver) and for the right HRVA along with the right pedicle width (inter-software). This means the overall prevalence might slightly differ between clinicians planning the craniocervical instrumentation. This might also partially account for differences between researchers across the continents, yet ethnic background and genetics constitute a more probable cause.

## Conclusions

A quarter of Central-European population have at least one HRVA, and about one-third of Central-Europeans have at least one narrow C2 pedicles (a quarter of potential screw insertion sites). There is no side predilection. In general, men appear to have larger C2 isthmi and C2 pedicles than women. Measurements can be consistently reproduced by various observers at varying intervals using different software. Thus, both HRVA and narrow C2 pedicles should be looked for at the planning stage prior to craniocervical instrumentation in order to choose the safest treatment option.

## Data Availability

The data that support the findings of this study are available from the corresponding author, TK, upon reasonable request.

## References

[1] Abou Madawi A, Solanki G, Casey AT, Crockard HA (1997). Variation of the groove in the axis vertebra for the vertebral artery. Implications for instrumentation. J Bone Joint Surg Br.

[2] Alshafai NS, Klepinowski T (2019) Extreme lateral approach to the craniovertebral junction: an update. In: Acta Neurochir Suppl. Springer-Verlag Wien:171–174. 10.1007/978-3-319-62515-7_25 10.1007/978-3-319-62515-7_25 30610319

[3] Alshafai NS, Klepinowski T (2019) The far lateral approach to the craniovertebral junction: an update. In: Acta Neurochir Suppl. Springer-Verlag Wien:159–164. 10.1007/978-3-319-62515-7_23 10.1007/978-3-319-62515-7_23 30610317

[4] Chazono M, Tanaka T, Kumagae Y, Sai T, Marumo K (2012). Ethnic differences in pedicle and bony spinal canal dimensions calculated from computed tomography of the cervical spine: a review of the English-language literature. Eur Spine J.

[5] Fujimori T, Le H, Hu SS (2015). Ossification of the posterior longitudinal ligament of the cervical spine in 3161 patients: A CT-Based Study. Spine (Phila Pa 1976).

[6] Klepinowski T, Cembik J, Sagan L (2020) Risk of the high-riding variant of vertebral arteries at C2 is increased over twofold in rheumatoid arthritis: a meta-analysis. Neurosurg Rev:1–6. 10.1007/s10143-020-01425-w 10.1007/s10143-020-01425-w PMC8338830 33106959

[7] Klepinowski T, Limanówka B, Sagan L (2020) Management of post-traumatic craniovertebral junction dislocation: A PRISMA-compliant systematic review and meta-analysis of casereports. Neurosurg Rev:1–10. 10.1007/s10143-020-01366-4 10.1007/s10143-020-01366-4 PMC8121741 32797319

[8] Klepinowski T, Pala B, Cembik J, Sagan L (2020). Prevalence of high-riding vertebral artery: a meta-analysis of the anatomical variant affecting choice of craniocervical fusion method and its outcome. World Neurosurg.

[9] Lofrese G, Cultrera F, Visani J, Nicassio N, Essayed WI, Donati R, Cavallo MA, de Bonis P (2019). Intraoperative Doppler ultrasound as a means of preventing vertebral artery injury during Goel and Harms C1-C2 posterior arthrodesis: Technical note. J Neurosurg Spine.

[10] Neo M, Matsushita M, Iwashita Y, Yasuda T, Sakamoto T, Nakamura T (2003). Atlantoaxial transarticular screw fixation for a high-riding vertebral artery. Spine (Phila Pa 1976).

[11] Neo M, Sakamoto T, Fujibayashi S, Nakamura T (2005). A safe screw trajectory for atlantoaxial transarticular fixation achieved using an aiming device. Spine (Phila Pa 1976).

[12] Vaněk P, Bradáč O, de Lacy P, Konopková R, Lacman J, Beneš V (2017). Vertebral artery and osseous anomalies characteristic at the craniocervical junction diagnosed by CT and 3D CT angiography in normal Czech population: analysis of 511 consecutive patients. Neurosurg Rev.

[13] Yao Q, Yin P, Khan K, Tsai TY, Li JS, Hai Y, Tang P, Li G (2018) Differences of the Morphology of Subaxial Cervical Spine Endplates between Chinese and White Men and Women. Biomed Res Int 2018. 10.1155/2018/2854175 10.1155/2018/2854175 PMC5838464 29675423

